# Lacosamide in Pediatric Status Epilepticus

**DOI:** 10.15844/pedneurbriefs-31-2-2

**Published:** 2017-02

**Authors:** Juan A. Piantino

**Affiliations:** 1 Department of Pediatrics, Section in Pediatric Neurology. Oregon Health and Science University, Portland, OR

**Keywords:** Status Epilepticus, Lacosamide, Children, Seizures

## Abstract

Investigators from Baylor College of Medicine studied the efficacy of lacosamide in pediatric status epilepticus (SE).

Investigators from Baylor College of Medicine studied the efficacy of lacosamide in pediatric status epilepticus (SE). This is a single center, retrospective observational study of the use of IV lacosamide in children with SE. Nine children with a mean age of 5.7 years (2 months to 16 years) were enrolled in the study. SE was defined as continuous seizure activity longer than 20 minutes or two or more recurrent seizures without regaining baseline level of awareness. Efficacy was defined as seizure freedom or more than 50% reduction of seizures within 24 hours of administering lacosamide.

The mean loading lacosamide dose was 8.7 mg/kg (7 received 10 mg/Kg). The total dose of lacosamide for the first 24 hours was 13.8 mg/Kg. Seven of nine patients (77.8%) experienced seizure improvement on lacosamide, and four patients (44.4%) became seizure free. Two patients continued to have SE within 24 hours of lacosamide administration. Bradycardia was observed in one patient. There were no other side effects documented in the study. [1]

COMMENTARY. SE is a medical emergency consisting of persistent or recurring seizures. It is a heterogeneous condition which can be divided into subtypes with multiple underlying etiologies [2]. Care involves both termination of seizures and identification and management of any underlying conditions. Despite the dramatic increase in the number of anticonvulsants over the past decade, the treatment of SE still revolves around older mainstay IV anticonvulsants, fosphenytoin or phenobarbital. However, the evidence behind the use of these drugs remains sparse [3], and to date there are no randomized trials done exclusively in children comparing the efficacy of those drugs versus newer agents. In addition, the use of these drugs is often associated with adverse effects such as respiratory depression and hypotension. The use of lacosamide in SE has been documented in several observational studies in the adult population [4-6]. In those studies, the percentage of patients who experienced seizure cessation was 40-50%. A previous observational study in children reported resolution of SE in 45% of patients [7]. Podddar et al., report a 77.7% response rate, with 44.4% becoming seizure free. Interestingly, lacosamide seems to be well tolerated, with bradycardia in one of the 9 study subjects being the only reported adverse effect. There are, however, several limitations to this study, which are recognized by the authors, the most significant one being sample size. In addition, several variables were not controlled for, including the dose of lacosamide, the administration of other anticonvulsants, and the timing of medication administration. Despite its limitations, this study adds to the accumulating body of literature suggesting that lacosamide is a promising and safe agent in the treatment of SE. Studies like this one are important as they highlight potential candidates for a very much needed randomized controlled trial of IV agents in pediatric SE.
